# Enhancing pix2pix with Swin Transformer for Cross Modal Brain CT-MR synthesis

**DOI:** 10.21203/rs.3.rs-7565545/v1

**Published:** 2025-09-25

**Authors:** Mario Verdicchio, Francesco Isgrò, Marco Salvatore, Marco Aiello

**Affiliations:** 1 IRCCS SYNLAB SDN, Naples, 80142, Italy; 2 Università degli Studi di Napoli Federico II, DIETI, Naples, 80133, Italy

## Abstract

Cross-modal medical image synthesis, such as generating a brain computed tomography (CT) from a magnetic resonance (MR) and vice versa, plays an increasingly crucial role in the management of cerebral diseases. Conventional CNN-based models, such as pix2pix, have demonstrated utility in this domain but are limited in capturing long-range dependencies and global anatomical context, often compromising fidelity.

This study introduces an enhanced image-to-image translation framework that replaces the standard U-Net generator in pix2pix with SwinUNETR, a transformer-based architecture. Leveraging hierarchical self-attention mechanisms, the model effectively captures both local and global features, enabling the synthesis of anatomically realistic images.

The framework was evaluated on CT-to-MR (sMR) and MR-to-CT (sCT) synthesis tasks using 2,091 paired CT and T1-weighted MR scans from public datasets (OASIS-3, SynthRAD2023) and an internal cohort of patients with neurodegenerative disorders. Quantitative metrics, including Multi-Scale Structural Similarity (MS-SSIM) and Peak Signal-to-Noise Ratio (PSNR), were used to benchmark performance against a pix2pix baseline.The proposed method consistently outperformed the baseline, achieving an MS-SSIM of 0.952 and a PSNR of 26.07 dB in sCT. In sMR, it achieved an MS-SSIM of 0.948 and a PSNR of 26.07 dB, while preserving gray–white matter contrast—an essential feature for the assessment of neurodegenerative diseases.

These results highlight the potential of Transformer-based architectures like SwinUNETR to advance high-fidelity cross-modal synthesis, particularly in neurological applications.

## Introduction

Recent advances in artificial intelligence (AI), particularly the release of increasingly powerful generative models, are profoundly impacting many aspects of daily life. Their applications range from creating photorealistic images and artistic content to accelerating scientific discovery in fields like drug development and materials science. Over the last decade, deep generative models including autoregressive models, Generative Adversarial Networks (GANs), Variational Autoencoders (VAEs), and more recently diffusion models^1–3^, which have emerged as the new state-of-the-art for high-fidelity image synthesis have reshaped content synthesis across domains such as imaging, video, audio, and text^4, 5^. Within this landscape, GANs^6^ remain a versatile framework for image-to-image translation. A significant milestone in this area is the Pix2Pix framework^7^, which demonstrated remarkable success in learning a mapping from an input image to a target image using paired training data. One of the most compelling applications for such techniques is in medical imaging, specifically for cross-modality synthesis (e.g., generating Computed Tomography (CT) scans from Magnetic Resonance (MR), or vice-versa)^8–10^

Building upon the success of the original model, which employs a U-Net-based generator with skip connections to fuse low- and high-level features, subsequent frameworks have introduced architectural refinements to address domain-specific challenges. In Pix2Pix, the U-Net generator typically follows an encoder–decoder structure composed of convolutional layers, batch normalization, and ReLU activations, paired with a patch-based discriminator that enforces both local and global consistency. While this design has proven effective for natural images, it faces significant challenges when applied to the high-dimensional, volumetric nature of medical imaging, where preserving fine anatomical details and ensuring global spatial coherence are critical. Several extensions of the Pix2Pix framework have been proposed to tackle medical image translation tasks, including MR-to-CT and CT-to-MR synthesis^11–15^. Attempts to adapt these models to the complexities of medical data have primarily followed two strategic lines. The first focuses on architectural refinements within the CNN paradigm. For example, some works have replaced the standard U-Net with more powerful convolutional backbones like Dense-UNet to improve feature extraction^15^, or have integrated attention modules such as CBAM into residual blocks to enhance the representation of salient features^11^. The second line of inquiry involves enhancing the objective function to better preserve critical structures. This includes designing complex loss cocktails that combine gradient loss for sharpness, VGG-based perceptual loss for edges, and even histogram-based losses specifically for bone generation^11^, or developing structural perceptual supervision methods to enforce alignment^14^. Other works have even shifted focus from architectural design to the training paradigm itself, proposing methods to handle misaligned data pairs, which are common in clinical practice^12^. While these approaches have yielded notable improvements, they are fundamentally bound by the properties of the convolutional architectures they rely on. The local nature of the convolutional kernel restricts the model’s ability to capture long-range spatial dependencies effectively, a critical requirement for maintaining global anatomical coherence in large volumetric scans.

The recognized limitations of CNNs in modeling global context have motivated a recent shift towards exploring Transformer-based models for medical image synthesis. This exploration has proceeded along two main fronts. In the domain of unpaired translation, pure Transformer architectures have been successfully employed; for instance, Zhu et al.^16^ integrated a Vision Transformer (ViT) into a CycleGAN framework to improve synthesis quality. In the context of paired translation, however, adoption has been more cautious, with proposed solutions often relying on hybrid strategies where a Transformer plays a limited, complementary role alongside a dominant CNN backbone^13^. While promising, these approaches leave the full potential of a pure, hierarchical Transformer within a paired framework largely unexplored. Significantly, this very limitation has been recently acknowledged. Li et al.^13^, for instance, explicitly identified the challenge of learning local and global relations simultaneously and proposed a hybrid model. Their solution applies a Transformer to the bottleneck of a low-resolution module to capture long-range context, while still relying on dense convolutional connections for high-resolution features.

To addres these limitations, an enhanced paired image-to-image translation framework is proposed, integrating a Transformer-based SwinUNETR architecture into the Pix2Pix setup. SwinUNETR^17^ combines the hierarchical Swin Transformer backbone^18^ with a U-Net–like encoder–decoder design, enabling efficient modeling of both local details and global context in volumetric medical images. The Swin Transformer^18^ employs a hierarchical structure with shifted windowing to capture long-range dependencies and multiscale relationships at reduced computational cost—capabilities crucial for maintaining structural consistency in medical image synthesis.

The core of the proposed framework is the replacement of the conventional CNN generator in Pix2Pix with the SwinUNETR architecture. This approach creates a powerful synergy: it harnesses the Transformer’s superior ability to model global context for long-range anatomical consistency while preserving the U-Net design’s proven strength in extracting fine-grained local features. To optimize the training process, this study also includes a systematic analysis of different loss function combinations. The complete method, which incorporates a tailored preprocessing pipeline to improve structural and intensity consistency, is robustly evaluated on two challenging cross-modality tasks: CT-to-MR synthetic MR (sMR) generation and MR-to-CT synthetic CT (sCT) generation.

## Methods

### Dataset

This study used a combination of publicly available and internal retrospective datasets, totaling 2,091 paired CT and T1-weighted MR images.

#### Public datasets.

Two public resources were included: the Open Access Series of Imaging Studies 3 (OASIS-3)^19^ and SynthRAD2023^20^. OASIS-3 is a longitudinal dataset providing clinical, neuropsychological, and multimodal neuroimaging data from older adults, including cognitively normal individuals as well as patients with mild cognitive impairment (MCI) and Alzheimer’s disease (AD). From OASIS-3, we selected 1,581 paired high-resolution T1-weighted MR and low-dose CT scans.

The SynthRAD2023 dataset^20^contains 1,080 paired CBCT–CT–T1wMR images covering abdominal and brain regions, originally released for the SynthRAD2023 Challenge. In this study, 180 T1wMR–CT pairs were used. These data were used exclusively for external validation and were not included in model training or internal validation. Unlike OASIS-3 and our internal datasets, which primarily feature healthy individuals and patients with neurodegenerative disorders, SynthRAD2023 mainly includes oncological patients, introducing a clinically distinct population.

#### Institutional dataset.

An institutional dataset consisting of 330 paired T1wMR–CT images was obtained from retrospective search within the IRCCS SYNLAB SDN institutional repository. It includes mainly patients with cognitive decline or dementia-related conditions. Regarding T1w sequence, the field of view (FOV) was set to 240 × 240 × 180 mm with isotropic voxels of 1 × 1 × 1 mm. Acquisition parameters included an echo time (TE) of 3.731 ms, a repetition time (TR) of 8.180 ms, and a flip angle of 8 degrees. No inversion time was applied. All CT scans were acquired using a Discovery 710 PET-CT scanner (GE Healthcare) operated in helical mode with a tube voltage of 95 kV and a tube current of 300 mA. All procedures were conducted in accordance with the Declaration of Helsinki and were approved by the local institutional y the Campania Ethics Committee (protocol 01/24) for the study *“DIADEMA: A new neuroradiological workflow for assisted diagnosis and management of dementia with artificial intelligence.”* . The requirement for informed consent was waived by the Campania Ethics Committee (protocol 01/24) owing to the design of the study.

#### Data split and rationale.

**Figure 1.**
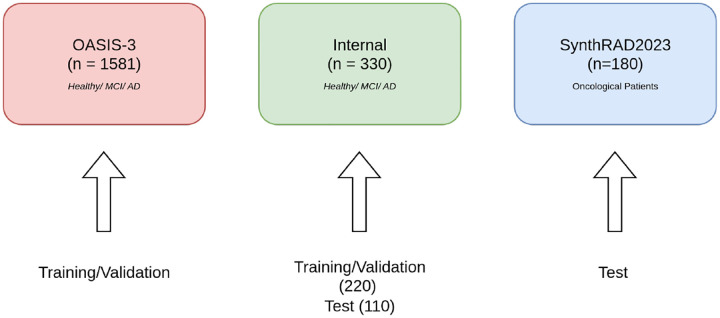
Figure 2. Datest composition and splitting schema

OASIS-3 and a part of the internal datasets (totaling 1,911 image pairs) were used for model training and validation. A portion of the internal dataset (110 paired images) and SynthRAD2023 (180 image pairs) served as an test set. The use of SynthRAD2023 dataset as test set, allow an evaluation of the model in the robustness under domain shift, given its distinct clinical composition and imaging characteristics and a comparison for MR→CT conversion . This separation ensures that performance estimates reflect the model’s ability to generalize beyond the specific distribution of the training data. The image distribution is visible in the Figure 1.

The pairing required an acquisition time window of ±180 days between modalities, a criterion consistently enforced across both public and our local (non-public) data sources.

### Data Preprocessing

To mitigate the challenges of anatomical misalignment and intensity inconsistency highlighted above, a dedicated preprocessing pipeline was designed, with distinct steps for MR and CT images. For MR images, the process began with N4 bias field correction to mitigate intensity non-uniformities and artifacts. Subsequently, non-brain tissue was removed using a skull stripping procedure based on the SynthStrip method^21^. The resulting skull-stripped MR images were then spatially normalized to the Montreal Neurological Institute (MNI152) standard space using the antsRegistrationSyNQuick function from the Advanced Normalization Tools (ANTs) software package^22^. Finally, an intensity normalization step was performed. The specific method was selected based on evaluations by Reinhold et al.^23^, inv0olving statistical normalization techniques similar to those described by Shinohara et al.^24^.

**Figure 3.**
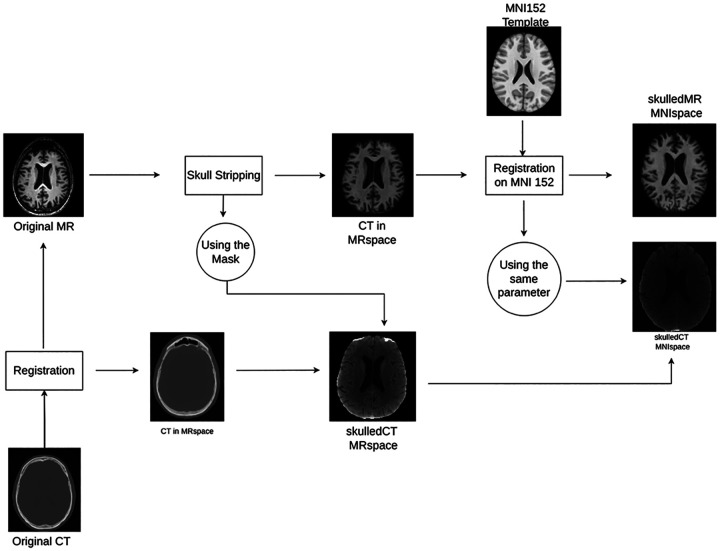
Registration Process

For Computed Tomography (CT) image preprocessing, the images were first co-registered to their corresponding skull-stripped T1-weighted MR images (in their native space, pre-MNI transformation) using the flirt function from the FSL software package^25^. Following co-registration, the CT images also underwent skull stripping, utilizing the brain mask created from the MR skull stripping process. Finally, the transformation parameters (e.g., deformation field) derived from the MR image normalization to MNI space were then applied to these co-registered, skull-stripped CT images to bring them into the same MNI standard space. A graphical visualization of this registration process is shown in Figure 3.

**Figure 4.**
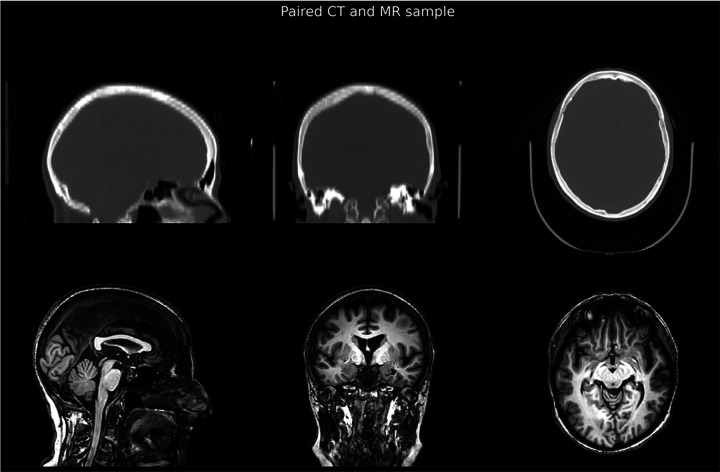
Example of Paired CT and MR images

For the CT→MR synthesis, the preprocessing pipeline utilized skull-stripped versions of both CT and MR images, co-registered and normalized into the MNI space to ensure precise anatomical alignment. Conversely, in the MR→CT synthesis pipeline, where preservation of the skull is required, a critical challenge arises from the fact that MR and CT images are often acquired with different field-of-views and patient poses: MR images frequently cover the head and neck region, while CT scans typically encompass only the head, this is visible in the Figure 4. To address this mismatch and ensure spatial correspondence limited to the head, a solid head mask that excludes the neck region was generated using a custom method.

This method involves combining information from both modalities (MR and CT) by applying intensity-based thresholding to isolate relevant anatomical structures in each image. The resulting binary masks undergo morphological closing operations to fill small holes and connect fragmented regions. Subsequently, the largest connected component is selected to ensure spatial coherence of the head mask. Finally, any remaining internal holes within this component are filled to produce a continuous solid mask representing the head alone. These methods is summarized in the algorithm

**Algorithm 1:** Solid Head Mask Generation from MR and CT Images

**Require:** MR image $I_{\text{MR}}$, CT image $I_{\text{CT}}$
**Ensure:** Binary solid head mask $M_{\text{head}}$
1: **Thresholding:**
$M_{\text{MR}}\leftarrow \left(I_{\text{MR}}>T_{\text{MR}}\right)$, $M_{\text{CT}}\leftarrow \left(I_{\text{CT}}>T_{\text{CT}}\right)$
2: **Morphological closing:** fill small holes and connect regions
$M_{\text{MR}}^{\text{closed}}\leftarrow \text{MorphologicalClosing}\left(M_{\text{MR}},r\right)$, $M_{\text{CT}}^{\text{closed}}\leftarrow \text{MorphologicalClosing}\left(M_{\text{CT}},r\right)$
3: **Intersection:**
$M_{\text{common}}\leftarrow M_{\text{MR}}^{\text{closed}}\cap M_{\text{CT}}^{\text{closed}}$
4: **Largest connected component:**
$M_{\text{largest}}\leftarrow \text{LargestConnectedComponent}\left(M_{\text{common}}\right)$
5: **Hole filling:**
$M_{\text{head}}\leftarrow \text{FillHoles}\left(M_{\text{largest}}\right)$
6: **return** $M_{\text{head}}$

This solid head mask is then used to restrict the region of interest during image synthesis, improving alignment and consistency when the skull is preserved, and also enabling removal of non-anatomical structures in the CT scans, such as the patient table.

### Model Architecture

The proposed image-to-image translation model is based on a Conditional Generative Adversarial Network (cGAN)^26^, leveraging the foundational Pix2Pix framework^7^. A cGAN comprises two distinct, competing neural networks: a generator (G) and a discriminator (D). The generator (G) is tasked with learning a mapping from an input image x (the conditioning input, e.g., a CT scan) to a plausible output image G(x) (e.g., a synthetic MR image). The objective of the generator is to produce images that are indistinguishable from real images to the discriminator. In the original Pix2Pix formulation, while a noise vector z is technically part of the cGAN definition, it is often omitted or added via dropout during training, simplifying the mapping to G:X→Y. The discriminator (D), conversely, learns to differentiate between real paired images (x, y) and synthetic paired images (x, G(x)), where y represents the ground truth output corresponding to input x. The discriminator aims to output a high probability for real pairs and a low probability for fake pairs. The adversarial training process is defined by the following objective function, typical of cGANs:

(1) $$L_{\text{cGAN}}=E_{x,y}[\text{log}D(x,y)]+E_{x,z}[\text{log}(1-D(x,G(x,z)))]$$

**Figure 5.**
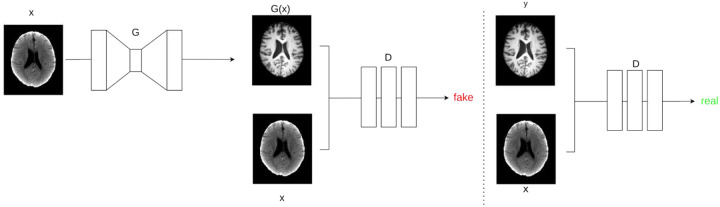
Conditional GAN training strategy. Example in CT→MR scenario

A schematic overview considering the task CTtoMR is proposed in fig 5

In the context of image-to-image translation, low-frequency information is better captured when an L1 penalty is added to the loss function^7^. The L1 term can be written as :

(2) $$L_{1}=E_{x,y,z}\|y-G(x,z)\|$$

and added to Equation 1.

To further enhance the quality and perceptual fidelity of the generated images, other terms were incorporated to loss terms objective function. Particularly, a Structural Similarity Index (SSIM) Loss^27^ was utilized. This loss specifically focuses on the perceptual quality of the images by evaluating the structural similarity between the generated outputs and the ground truth. It is particularly effective in preserving textures and structural information, which enhances the visual realism and coherence of the synthesized images. The SSIM metric between two images x and y is defined as:

(3) $$\text{SSIM}(x,y)=\frac{\left(2\mu_{x}\mu_{y}+C_{1}\right)\left(2\sigma_{xy}+C_{2}\right)}{\left(\mu_{x}^{2}+\mu_{y}^{2}+C_{1}\right)\left(\sigma_{x}^{2}+\sigma_{y}^{2}+C_{2}\right)}$$

(4) $${ℒ}_{\text{SSIM}}=1-\text{SSIM}(x,y)$$

Where :

- $\mu_{x}$ and $\mu_{y}$ are the means of the images x and y; In our model, the generator
- $\sigma_{x}^{2}$ and $\sigma_{y}^{2}$ are the variances of x and y;
- $\sigma_{\text{xy}}$ is the covariance between x and y;
- $C_{1}$ and $C_{2}$ are constants to stabilize the denominator.

Furthermore, to comprehensively improve the perceptual fidelity and visual realism of the generated images, a perceptual loss term^28^ was also integrated into the objective function.

Perceptual loss^29^ is a type of loss function frequently employed in deep learning for image generation and translation tasks to produce more visually realistic results. Unlike traditional pixel-wise losses (such as L1 or Mean Squared Error), which directly compare raw pixel values, perceptual loss operates by measuring differences in high-level feature spaces. This approach aims to overcome the limitations of traditional metrics, which often fail to capture the structural and semantic similarities perceived by the human eye.

The strength of perceptual loss lies in its ability to leverage the hierarchical representations learned by pre-trained deep neural networks. Instead of directly comparing pixels, it compares the “feature maps” extracted from intermediate layers of these networks. The underlying idea is that if two images produce similar features within a deep layer of a network trained for visual tasks, then they are likely to be perceived as structurally and semantically similar by humans as well. pecifically for this study, the pre-trained model used for extracting these features is MedicalNet by Chen et al.^30^. This model was chosen because it has been extensively trained on a large dataset of 3D medical images, making its extracted features particularly relevant for analyzing data within this specialized domain. The implementation of the perceptual loss is provided by the MONAI framework^31^, which integrates the capability to extract and compare these deep features for a more perceptually accurate evaluation. The perceptual loss ($L_{P}$) can be formulated as the sum of L1 distances between the feature representations $\phi_{j}(\cdot )$ extracted from N selected layers j of the pre-trained network:

(5) $$L_{P}=Ex,y,z\left[\sum j=1^{N}\frac{1}{W_{j}H_{j}C_{j}}{\left|\phi_{j}(y)-\phi_{j}(G(x,z))\right|}_{1}\right]$$

where y is the ground truth image, x represents the input condition, and z is a noise vector fed to the generator $\text{G}.\phi_{j}(I)$ is the activation of the j-th chosen layer of the pre-trained network (provided by MONAI) when processing image I, and $W_{j},H_{j},C_{j}$ are the width, height, and number of channels of the feature map at layer j, respectively, used here as the normalization factor M

The final objective function, incorporating the cGAN loss, L1 loss, SSIM loss, and the perceptual loss, is therefore:

(6) $$L_{\text{total}}=L_{\text{cGAN}}+\lambda_{L1}L_{L1}+\lambda_{\text{SSIM}}L_{\text{SSIM}}+\lambda_{P}L_{P}$$

where $\lambda_{L1},\lambda_{\text{SSIM}}$, and $\lambda_{P}$ are weighting parameters that control the relative importance of each loss term.

#### Generator

**Figure 6.**
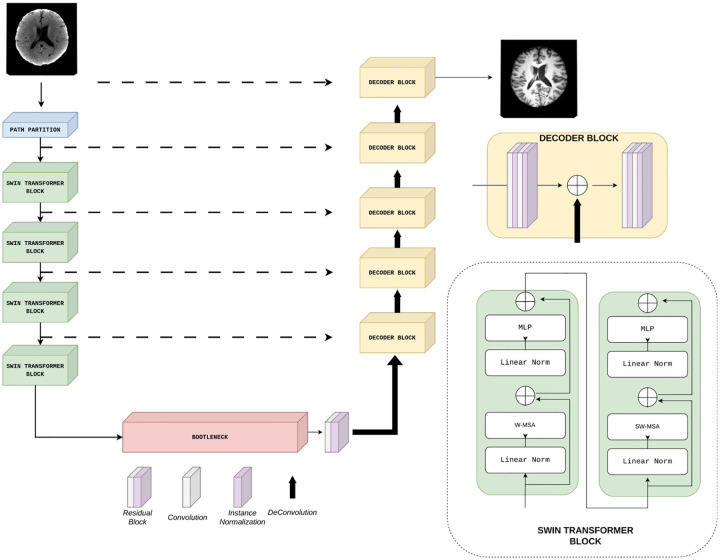
Generator schema based on SwinUNETR architecture

In the proposed model, the generator is defined as a Swin UNETR architecture^17^ that leverages the strengths of the Swin Transformer encoder^18^ to extract multi-resolution features across five scales. A schematic overview of this architecture is shown in Figure 6.

It follows an encoder–decoder design, where the encoder comprises a hierarchy of Swin Transformer stages that progressively partition the input into non-overlapping windows, apply self-attention within shifted windows, and downsample the resulting feature maps to capture increasingly abstract representations at multiple scales. The decoder reconstructs the output by progressively upsampling the feature maps. Skip connections from the encoder ensure that spatial details and structural information are preserved throughout the reconstruction process. By combining local feature modeling within windows and global context aggregation through window shifting, the architecture effectively captures both fine-grained details and long-range dependencies, which are essential for preserving structural coherence and enhancing the quality of the generated images.

The objective of the generator is defined as follows:

(7) $$\text{arg}\;\underset{G}{\text{min}}{ℒ}_{\text{cGAN}}(G,D)+\lambda_{1}{ℒ}_{1}(G)+\lambda_{\text{SSIM}}{ℒ}_{\text{SSIM}}(G)+\lambda_{P}L_{P}$$

As a baseline, a fully convolutional U-Net generator^32^ was implemented, which adapts the architecture from the original Pix2Pix paper^7^ for 3D volumetric data. The model features a symmetric encoder-decoder architecture built upon a series of nested blocks.

The encoder path consists of five downsampling stages. Each stage uses a 3×3 strided convolution (with a stride of 2) to halve the spatial dimensions and progressively double the number of feature channels, starting from a base of 64. This operation is followed by a Batch Normalization layer and a LeakyReLU activation.

The decoder path mirrors the encoder with five upsampling stages. Upsampling is performed via 4×4 transposed convolutions (with a stride of 2). Crucially, skip connections concatenate the feature maps from the encoder with the corresponding decoder stages to preserve low-level spatial details. Each upsampling block includes the transposed convolution, Batch Normalization, a ReLU activation, and optional Dropout layers.

#### Discriminator

For the discriminator, we employ PatchGAN architecture originally proposed by Isola et al.^7^, which divides input images into smaller patches and classifies each one independently before averaging the results. While a 70 × 70 patch size is conventionally used to mitigate tiling artifacts, insights derived from MedGAN^33^ motivated us to experiment with a smaller patch size. Their findings indicate that a 16 × 16 patch configuration yields sharper and more detailed outputs, eliminating tiling artifacts without compromising performance. This design is implemented through two convolutional layers with 64 and 128 spatial filters, each followed by batch normalization and Leaky-ReLU activation functions. The obejctive of the discriminator was defined as follow:

(8) $$\text{arg}\;\underset{D}{\text{max}\;}{ℒ}_{\text{cGAN}}(G,D)$$

#### Training Settings

The primary dataset for model development was partitioned at the patient level to create independent training and validation sets. A randomized 80/20 split was used, allocating 80% of subjects to the training set and the remaining 20% to the validation set. This patient-wise division prevents data leakage and ensures that the validation set provides an unbiased estimate of generalization performance during training. Where multiple source datasets were pooled, this random partitioning also aimed to maintain a representative distribution of data sources across both sets. The validation dataset was employed for model selection, based on the SSIM evaluated during training. All models, the proposed method and both baselines, were trained under identical conditions to ensure a fair comparison. The training protocol was as follows:

- Loss weights were chosen ($\lambda_{1}=50;\lambda_{\text{SSIM}}=25;\lambda_{P}=25$)
- Optimizer: Adam, with momentum parameters configured to β1=0.5 and β2=0.999.
- Epochs: Training was conducted for a total of 100 epochs.
- Learning Rate: An initial learning rate of 0.0002 was used. This rate was maintained for the first 50 epochs and was then linearly decayed to zero over the final 50 epochs.
- Initialization: All network weights were initialized from a Gaussian distribution.

### Performance Evaluation

To ensure a comprehensive validation, the proposed model was evaluated against a standard Pix2Pix implementation using a U-Net generator. An ablation study was also conducted to assess the impact of different loss functions on synthesis quality. To further assess the model’s robustness and generalization, it was validated on a proprietary internal dataset of 110 patient cases and the public SynthRAD2023 challenge dataset.

The quantitative evaluation across these experiments was performed using several Image Quality Metrics (IQM), which are designed to mimic human perception. For this study, the following measures were employed:

- Structural Similarity Index
- Multi-Scale Structural Similarity Index
- Mean Square Error
- Peak Signal-to-Noise Ratio

The Structural Similarity (SSIM) Index^27^, is a perceptual metric that assesses image quality degradation resulting from various processing techniques or data transmission losses. It operates by calculating an index on different windows of an image. For two windows x and y of size N × N, the SSIM is computed following the formula 3.

Building upon the single-scale SSIM, the Multi-Scale Structural Similarity (MS-SSIM) index^27^ was also employed. MS-SSIM provides a more comprehensive perceptual quality assessment by combining structural, luminance, and contrast comparisons across multiple image scales. This multi-scale approach often correlates better with subjective human quality judgments, particularly for distortions that manifest differently across resolutions.

Mean Square Error (MSE)^34^ is a fundamental metric in image quality assessment, representing the second moment of error. It calculates the average squared difference between corresponding pixels in the original and distorted images. MSE between two images such as g(x,y) and $\overset{ˆ}{g}(\text{x},\text{y})$ is defined as

(9) $$\text{MSE}=\frac{1}{\text{MN}}\sum_{n=0}^{M}\sum_{m=1}^{N}[\overset{ˆ}{g}(n,m)-g(n,m){]}^{2}$$

Peak Signal-to-Noise Ratio (PSNR)^34^, derived from MSE, quantifies the ratio between the maximum possible signal power and the power of distorting noise that affects image quality. It is typically expressed in decibels (dB). PSNR is expressed as:

(10) $$\text{PSNR}=10{\text{log}}_{10}\frac{\left({\text{peakval}}^{2}\right)}{\text{MSE}}$$

## Results

**Figure 7.**
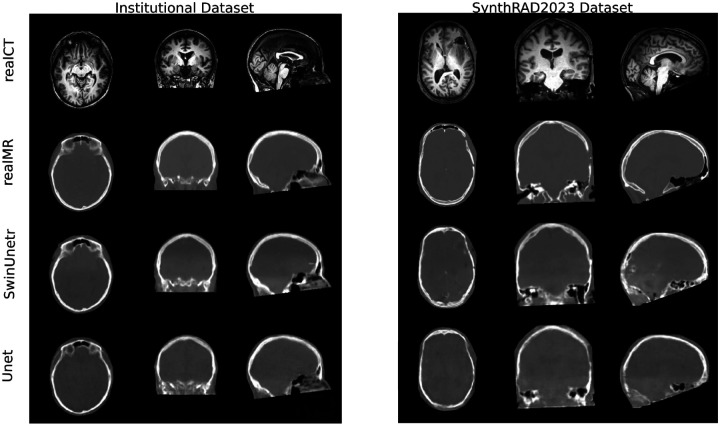
MR→CT example results

**Figure 8.**
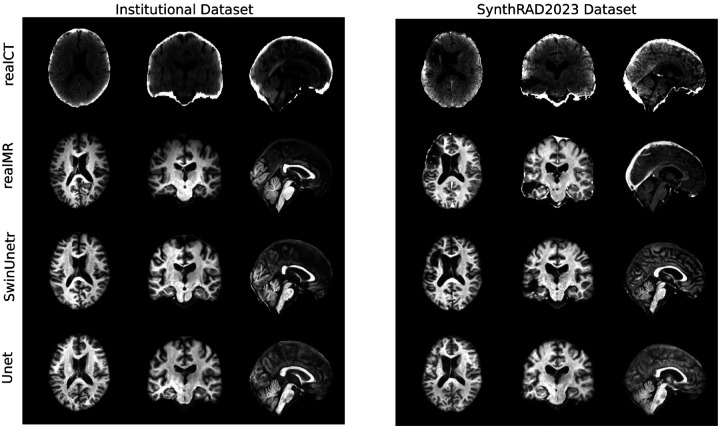
CT→MR example results

Following the experimental protocol outlined previously, a comprehensive evaluation was performed. The performance of the proposed SwinUNETR-based model is first compared against the U-Net baseline on the primary test set, followed by the findings from the loss function ablation study. The section concludes with an analysis of the model’s generalization capabilities on the external datasets. Qualitative evaluation of generated images is presented in Figures 7 and 8.

### MR-to-CT

As shown in Table 1, the UNet baseline was consistently outperformed by the SwinUNETR-based model across nearly all configurations. The best results on the internal test set were achieved using the combination of $L_{1}$ + perceptual + structural loss, with an MS-SSIM of 0.952 and a PSNR of 26.074 dB. The highest performance obtained by the UNet configuration (L1 + structural) reached an MS-SSIM of 0.922 and a PSNR of 24.136 dB, indicating a notable improvement in perceptual quality and structural fidelity when SwinUNETR was employed. Similar trends were observed on the SynthRAD2023 dataset, further reinforcing the robustness of the approach. Visual inspection (Figure 7) confirms that sharper anatomical details, particularly in the skull, were generated by SwinUNETR, whereas outputs produced by UNet appeared slightly blurrier, in agreement with the MS-SSIM scores. However, a closer qualitative analysis of the SynthRAD2023 dataset revealed a less pronounced improvement. As shown in Figure 7, a higher incidence of visual artifacts and a reduced level of anatomical coherence were observed in the synthetic CT images compared to the internal test set. Fine structures were found to be less defined, and the overall image texture was perceived as less realistic. Consequently, while numerical metrics remained relatively high, the perceived visual quality was affected, emphasizing the importance of integrating both quantitative and qualitative evaluations, particularly for external datasets.

### CT-to-MR

The advantages of SwinUNETR were even more pronounced in the CT-to-MR task (Table 2). Using the full $L_{1}$ + percpetual + structural loss, SwinUNETR achieved an MS-SSIM of 0.912 and a PSNR of 24.512 dB, outperforming the UNet baseline (MS-SSIM 0.875, PSNR 23.283 dB). Qualitative results (Figure 8) show SwinUNETR better preserves the contrast and boundaries between gray and white matter. Gyri and sulci are more clearly defined, producing synthetic MR images that are anatomically closer to the ground truth, whereas UNet outputs show less distinct tissue boundaries and reduced fine structural detail.

In summary, both the quantitative and qualitative analyses robustly indicate that replacing the standard CNN-based generator with the SwinUNETR architecture leads to a marked improvement in synthetic image quality. This enhancement is most evident in the perceptual metrics (MS-SSIM) and visual fidelity, suggesting that the generated images are both pixel-wise and structurally accurate.

## Discussion

The results of this study robustly demonstrate the superiority of the Swin UNETR architecture as a generator within a Pix2Pix framework for medical image synthesis, compared to a traditional CNN-based UNet^35^. This performance enhancement, observed both quantitatively through metrics like MS-SSIM and PSNR and qualitatively through visual inspection, is evident in both the MR→CT and CT→MR translation tasks.

This approach is aligned with a growing trend in the literature exploring hybrid or Transformer-based architectures. For instance, ResViT^36^, HMSS-Net^13^ and Trans-GAN^37^ have both demonstrated how combining convolutional modules for local feature extraction with the global attention of Transformers leads to superior performance, confirming that the architectural choice adopted here is both well-founded and promising. In contrast, purely Transformer-based approaches, such as Zhu et al.^16^, have been successfully applied in the domain of unpaired translation, highlighting the representational power of attention mechanisms. In this context, the present study leverages both local feature modeling and global contextual understanding, benefiting from the strengths of both paradigms.

The primary advantage of Swin UNETR lies in its ability to model long-range dependencies and capture the global context of the image through its Transformer-based self-attention mechanism^17, 18^. Unlike CNNs, which operate on local receptive fields, Transformers can correlate distant regions of the input image^38^. This capability is particularly beneficial in anatomy, where global structural coherence (e.g., skull symmetry or brain morphology) is crucial. The qualitative evidence, which shows sharper anatomical details and better-defined tissue boundaries (Figures 7 and 8), supports the hypothesis that this global context awareness translates into more realistic and faithful synthetic images.

The model’s efficacy was further enshanced by the multi-component loss function. The ablation study revealed a critical synergy between the $L_{1}$, perceptual, structural, and adversarial losses. The adversarial component, a cornerstone of GAN frameworks such as Pix2Pix and CycleGAN^39^, encouraged the generation of realistic images. The perceptual loss, consistent with studies like SOUP-GAN^40^, improved sharpness and fine details, mitigating the blurriness typically associated with pixel-wise losses. Finally, the inclusion of a structural loss (SSIM), as explored in works on contrast-weighted SSIM^41^, aligned the training process with clinically relevant evaluation metrics. This multi-objective approach, similar to frameworks like MedGAN^33^, provided a balanced training signal, combining numerical accuracy with perceptual realism and clinical interpretability.

As emphasized in the introduction, one of the main challenges of supervised synthesis lies in the requirement for spatially aligned data. To address this, a rigorous preprocessing pipeline based on image registration was implemented. The high anatomical fidelity achieved in the synthetic images provides indirect evidence of the effectiveness of this strategy, since misregistration would likely have introduced artifacts. Nevertheless, this dependence on paired and registrable data represents a limitation, restricting applicability in unpaired scenarios where unsupervised approaches (e.g., CycleGAN-like models) remain the only viable solution.

A deeper analysis of the qualitative results also reveals limitations in terms of generalizability. On the SynthRAD 2023 test set, despite competitive quantitative scores, the generated CT images appeared visually more degraded and less anatomically coherent. This discrepancy is likely attributable to a domain shift between the training data and the SynthRAD cohort, due to variations in scanner type, acquisition protocol, contrast, resolution, or anatomical variability. Similar mismatches between numerical metrics and perceptual quality under domain shift have been reported in other cross-modality synthesis studies^42, 43^, underscoring the need for more semantically grounded evaluation metrics and the importance of rigorous qualitative analysis alongside standard measures such as PSNR and SSIM.

This study demonstrated that integrating the Swin UNETR architecture into a Pix2Pix framework substantially improves medical image synthesis compared to traditional CNN-based generators.Both quantitative metrics and visual inspection of representative examples indicate that the proposed Swin UNETR-based generator better preserves fine anatomical structures, maintains global spatial coherence, and captures subtle tissue contrasts compared to conventional U-Net–based generators for MR→CT and CT→MR translation tasks. It should be noted that the qualitative assessment was based on visual examination of selected images and not on a formal expert-based evaluation.

Nevertheless, several considerations and limitations must be acknowledged. The proposed framework relies on paired and registered data, which is an intrinsic characteristic of the Pix2Pix paradigm. While this requirement could be satisfied in our study, it may limit the generalizability of the approach to other clinical scenarios where paired acquisitions are not readily available.

Beyond this methodological consideration, practical challenges were observed in our experiments. Notably, the qualitative degradation observed on the SynthRAD 2023 dataset highlights the difficulties posed by domain shift, emphasizing the need for improved generalization across diverse imaging protocols and patient populations. Furthermore, the discrepancy between conventional quantitative metrics, such as PSNR and SSIM, and visual quality underscores their inadequacy in fully capturing clinical validity. Future work will address these challenges by extending the current supervised framework toward more flexible paradigms. Particular emphasis will be placed on exploring diffusion-based generative models, which may combine the representational power of Transformers with enhanced stability and data efficiency. Moreover, downstream clinical evaluations, such as assessing the utility of synthetic CT for radiotherapy planning, and the adoption of perceptually aligned metrics and expert-based qualitative assessments, will be essential to establish the clinical reliability of synthetic imaging methods.

## Figures and Tables

**Table 1.** Metrics for MR→CT task. Rows in light blue indicate the complete loss setting.

Loss Function	SWINUNETR				UNET			
	MAE ↓	MM-SSIM ↑	PSNR ↑	SSIM ↑	MAE ↓	MM-SSIM ↑	PSNR ↑	SSIM ↑
Institutional								
L1	0.032 ± 0.006	0.894 ± 0.020	22.654 ± 1.221	0.832 ± 0.015	0.031 ± 0.004	0.851 ± 0.016	22.730 ± 0.800	0.780 ± 0.007
L1 + PERCEPTUAL	0.040 ± 0.008	0.877 ± 0.022	21.163 ± 1.252	0.795 ± 0.017	0.033 ± 0.005	0.845 ± 0.016	22.165 ± 0.923	0.785 ± 0.007
L1 + PERCEPTUAL + STRUCTURAL	0.024 ± 0.004	0.909 ± 0.018	24.512 ± 1.284	0.856 ± 0.014	0.029 ± 0.005	0.875 ± 0.018	23.282 ± 1.144	0.812 ± 0.009
L1 + STRUCTURAL	0.026 ± 0.006	0.909 ± 0.021	24.215 ± 1.454	0.851 ± 0.016	0.030 ± 0.006	0.886 ± 0.023	22.969 ± 1.347	0.824 ± 0.012
SynthRAD2023								
L1	0.031 ± 0.005	0.852 ± 0.035	22.868 ± 1.249	0.809 ± 0.018	0.032 ± 0.005	0.812 ± 0.032	22.506 ± 1.041	0.772 ± 0.010
L1 + PERCEPTUAL	0.036 ± 0.007	0.852 ± 0.036	22.217 ± 1.454	0.779 ± 0.030	0.035 ± 0.006	0.815 ± 0.030	21.974 ± 1.148	0.780 ± 0.011
L1 + PERCEPTUAL + STRUCTURAL	0.029 ± 0.007	0.846 ± 0.054	23.098 ± 1.736	0.826 ± 0.024	0.029 ± 0.006	0.833 ± 0.043	23.186 ± 1.454	0.804 ± 0.013
L1 + STRUCTURAL	0.029 ± 0.007	0.848 ± 0.048	23.238 ± 1.706	0.820 ± 0.023	0.033 ± 0.008	0.832 ± 0.051	22.346 ± 1.715	0.806 ± 0.019

**Table 2.** Metrics for CT→MR task. Rows in light blue indicate the complete loss setting.

Loss Function	SWINUNETR				UNET			
	MAE ↓	MM-SSIM ↑	PSNR ↑	SSIM ↑	MAE ↓	MM-SSIM ↑	PSNR ↑	SSIM ↑
Institutional								
L1	0.023 ± 0.008	0.931 ± 0.020	26.064 ± 3.050	0.925 ± 0.019	0.030 ± 0.012	0.927 ± 0.020	24.655 ± 3.423	0.840 ± 0.064
L1 + PERCEPTUAL	0.023 ± 0.009	0.936 ± 0.019	26.026 ± 2.983	0.912 ± 0.027	0.038 ± 0.013	0.902 ± 0.028	23.507 ± 3.547	0.716 ± 0.093
L1 + PERCEPTUAL + STRUCTURAL	0.024 ± 0.009	0.948 ± 0.017	26.074 ± 3.347	0.934 ± 0.023	0.031 ± 0.011	0.920 ± 0.020	24.237 ± 3.007	0.836 ± 0.060
L1 + STRUCTURAL	0.029 ± 0.013	0.949 ± 0.017	25.130 ± 4.149	0.939 ± 0.021	0.030 ± 0.011	0.922 ± 0.020	24.136 ± 3.122	0.875 ± 0.045
SynthRAD2023								
L1	0.024 ± 0.009	0.835 ± 0.036	24.668 ± 2.091	0.855 ± 0.025	0.024 ± 0.011	0.846 ± 0.036	25.005 ± 2.535	0.839 ± 0.045
L1 + PERCEPTUAL	0.023 ± 0.010	0.849 ± 0.037	24.972 ± 2.312	0.863 ± 0.027	0.029 ± 0.012	0.837 ± 0.037	24.484 ± 2.469	0.720 ± 0.098
L1 + PERCEPTUAL + STRUCTURAL	0.024 ± 0.008	0.841 ± 0.035	24.550 ± 1.822	0.864 ± 0.025	0.025 ± 0.011	0.842 ± 0.036	24.616 ± 2.380	0.841 ± 0.036
L1 + STRUCTURAL	0.023 ± 0.011	0.857 ± 0.036	24.939 ± 2.472	0.873 ± 0.024	0.024 ± 0.011	0.849 ± 0.035	25.032 ± 2.555	0.852 ± 0.035
